# TLR7 activation in epilepsy of tuberous sclerosis complex

**DOI:** 10.1007/s00011-019-01283-3

**Published:** 2019-09-11

**Authors:** Alan A. Dombkowski, Daniela Cukovic, Shruti Bagla, McKenzie Jones, Joseph A. Caruso, Harry T. Chugani, Diane C. Chugani

**Affiliations:** 1 Department of Pediatrics, Wayne State University School of Medicine, Detroit, MI USA; 2 Institute of Environmental Health Sciences, Wayne State University, Detroit, MI 48201 USA; 3 Katzin Diagnostic and Research PET/MR Center, Nemours/Alfred I. duPont Hospital for Children, Wilmington, DE USA; 4 Department of Neurology, Thomas Jefferson University, Philadelphia, PA USA; 5 Departments of Communication Sciences and Disorders, and Chemistry and Biochemistry, University of Delaware, Newark, DE USA; 6 Children’s Hospital of Michigan, Clinical Pharmacology Room 3L22, 3901 Beaubien Blvd., Detroit, MI 48201 USA

**Keywords:** Neuroinflammation, Epilepsy, Tuberous sclerosis complex, Toll-like receptor, MicroRNA, AMT-PET

## Abstract

**Background:**

Neuroinflammation and toll-like receptors (TLR) of the innate immune system have been implicated in epilepsy. We previously reported high levels of microRNAs miR-142-3p and miR-223-3p in epileptogenic brain tissue resected for the treatment of intractable epilepsy in children with tuberous sclerosis complex (TSC). As miR-142-3p has recently been reported to be a ligand and activator of TLR7, a detector of exogenous and endogenous single-stranded RNA, we evaluated TLR7 expression and downstream IL23A activation in surgically resected TSC brain tissue.

**Methods:**

Gene expression analysis was performed on cortical tissue obtained from surgery of TSC children with pharmacoresistent epilepsy. Expression of TLRs 2, 4 and 7 was measured using NanoString nCounter assays. Real-time quantitative PCR was used to confirm TLR7 expression and compare TLR7 activation, indicated by IL-23A levels, to levels of miR-142-3p. Protein markers characteristic for TLR7 activation were assessed using data from our existing quantitative proteomics dataset of TSC tissue. Capillary electrophoresis Western blots were used to confirm TLR7 protein expression in a subset of samples.

**Results:**

TLR7 transcript expression was present in all TSC specimens. The signaling competent form of TLR7 protein was detected in the membrane fraction of each sample tested. Downstream activation of TLR7 was found in epileptogenic lesions having elevated neuroinflammation indicated by clinical neuroimaging. TLR7 activity was significantly associated with tissue levels of miR-142-3p.

**Conclusion:**

TLR7 activation by microRNAs may contribute to the neuroinflammatory cascade in epilepsy in TSC. Further characterization of this mechanism may enable the combined of use of neuroimaging and TLR7 inhibitors in a personalized approach towards the treatment of intractable epilepsy.

## Introduction

A growing body of evidence suggests that a ‘vicious cycle’ exists between seizures and neuroinflammation in some forms of epilepsy. In this scenario, a central nervous system insult due to injury, genetic alteration, or infection causes an initial onset of seizures that triggers a neuroinflammatory cascade, which further contributes to recurrent seizures [1]. A clinical biomarker associated with neuroinflammation and having nearly 100% specificity for identifying epileptogenic lesions is positron emission tomography (PET) imaging using the tracer α-methyl-l-tryptophan (AMT) [2]. Cellular uptake of AMT is increased in tissue where the kynurenine pathway (KP) of tryptophan metabolism is activated by neuroinflammatory signaling, primarily through induction of indoleamine 2,3-dioxygenase (IDO1) and/or tryptophan 2,3-dioxygenase (TDO2), rate-limiting enzymes of the pathway [3–5].

Tuberous sclerosis complex (TSC) arises due to genetic mutations that cause lesions in a number of organs, including the brain (tubers). Some cortical tubers trigger the onset of seizures (epileptogenic), while others do not. We recently reported elevation of several microRNAs in epileptogenic tubers from TSC patients [6]. Among these microRNAs, expression of miR-142-3p and miR-223-3p was strikingly correlated with AMT uptake, suggesting neuroinflammatory relevance. Studies have shown intercellular signaling mediated by cellular secretion of miR-142-3p in exosomes. A recent report found that miR-142-3p is a ligand and potent activator of the TLR7 receptor [7]. Additionally, others have shown that TLR7 can induce IDO1 and the KP [8]. Together, these findings suggest that the TLR7 receptor may be activated by the high level of miR-142-3p found in AMT-hot tissue, and miR-142-3p may play a role in neuroinflammation and KP induction in epileptogenic lesions. Currently, the role of TLR7 in epilepsy is unknown. Here, we sought to determine if TLR7 is expressed in TSC brain tissue and if miR-142-3p levels are associated with the receptor’s activation as indicated by IL-23A expression, a marker of TLR7 activation [9]. We also measured expression of TLRs 2 and 4, which have been reported increased in TSC and can act synergistically with TLR7 [10, 11]. This study has considerable translational relevance because TLR7 can be inhibited with existing drugs that are used to treat several autoimmune disorders.

## Methods

### Tissue specimens

Table 1 provides demographic data for each tissue specimen analyzed in this study and the assays performed on each specimen. Seizure onset and AMT status were determined as described in [6]. The amount of tissue available for each specimen is typically very limited and restricts the number of assays available for some samples. Informed consent was obtained from all participants, and the study was completed under Institutional Review Board #043515MP4E.

**Table 1:** Patient demographics and specimen details

Sample	Age	Gender	Hemisphere	Mutation	Location	Group	Lesion	Assays
L1412RK-C	2 years	M	Right	TSC2	Frontal	Normal	Non-tuber	a–d
I1710AC-B	5 years	M	Left	TSC2	Frontal parietal	Normal	Non-tuber	a–d
I1914MC-C	13 months	F	Left	TSC2	Parietal	Normal	Non-tuber	a–d
E0214IM-A	3 years	M	Right	TSC2	Parietal	Normal	Non-tuber	a–d
E0214IM-B	3 years	M	Right	TSC2	Frontal	Normal	Non-tuber	e
K2213NN-A	6 years	M	Right	TSC2	Occipital	Normal	Non-tuber	e
92804-B	7.5 years	F	Left	Unknown	Frontal	NC	Tuber	a–d*
83002-A	3 years	F	Left	TSC2	Temporal	NC	Tuber	a–e*
L1412RK-B	2 years	M	Right	TSC2	Frontal	NC	Tuber	a–d*
I1710AC-C	5 years	M	Left	TSC2	Frontal	NC	Tuber	a–d*
81603-A	8 years	M	Left	TSC2	Temporal	NC	Tuber	a–d*
G2710CC-A	2 years	M	Right	TSC2	Temporal	NC	Tuber	e
G2710CC-B	2 years	M	Right	TSC2	Occipital	OC	Tuber	a–e*
I1710AC-A	5 years	M	Left	TSC2	Frontal	OC	Tuber	a–d*
92804-A	7.5 years	F	Left	Unknown	Frontal	OC	Tuber	a–d*
J1513AB-B	11 years	F	Left	TSC2	Frontal parietal	OC	Tuber	a–d*
83002-D	3 years	F	Left	TSC2	Temporal	OC	Tuber	e
F0508-B	9 months	M	Left	TSC2	Occipital	OH	Tuber	d, e
L1412RK-A	2 years	M	Right	TSC2	Frontal	OH	Tuber	a–d*
81603-B	8 years	M	Left	TSC2	Frontal	OH	Tuber	a–d*
F0508-A	9 months	M	Left	TSC2	Central frontal	OH	Tuber	a–d*
83002-B	3 years	F	Left	TSC2	Parietal	OH	Tuber	a–e*

a: miR-142-3p qPCR, b: IL23A qPCR, c: TLR7 qPCR, d: TLR NanoString, e: Western

*miR-142-3p qPCR data from [6]

### Real-time quantitative PCR (qPCR) of TSC specimens

Gene expression analysis was performed using surgically resected TSC tissue, as indicated in Table 1. Of these, miR-142-3p data from a previous study were used for 13 specimens [6], representing three types of TSC tubers: seizure onset/AMT-hot (OH), onset/AMT-cold (OC), and non-onset/AMT-cold (NC), where “hot” indicates elevated AMT uptake detected in clinical AMT-PET imaging and “onset” indicates epileptogenic activity detected by clinical electrocorticography. These specimens were supplemented by four additional control samples of non-tuber cortical tissue from TSC patients and several additional tuber specimens to enable Western blotting.

Gene expression qPCR for TLR7 and IL-23A was performed as previously described [6], using 2XPowerUp SYBRGreen master mix and the following primers:

- TLR7/forward: 5′GCTGATCTTGGCACCTCTC3′
- TLR7/reverse: 5′TGTCCACATTGGAAACACCATT3′
- IL-23A/forward: 5′CTCAGTGCCAGCAGCTTTC 3′
- IL-23A/reverse: 5′CCACACTGGATATGGGGAAC 3′

MicroRNA qPCR was performed for miR-142-3p on the four control samples, as described in [6], and miR-142-3p data for the other 13 specimens were utilized from [6].

### NanoString expression assay of TLRs

Measurement of TLR 2, 4, and 7 levels was performed using the nCounter human neuroinflammation panel v1.0. For each sample as indicated in Table 1, 100 ng of total RNA was used as input to the hybridization, performed at 65 °C for 17 h. Data were background subtracted by the geometric mean of negative controls and normalized by the geometric mean of positive controls using nSolver v4.0.

### Capillary electrophoresis immunoblotting of TLR7

Membrane and cytosolic protein fractions were isolated from frozen tissue using the Qiagen Qproteome Cell Compartment kit [12] and approximately 20–30 mg of frozen brain tissue. The brain tissue was disrupted in 500-μl lysis buffer supplemented with Protease Inhibitor Solution using a TissueRuptor (Qiagen) for 5 s at the lowest speed followed by QIAshredder homogenizer step. Subcellular fractionation of the brain tissue samples was conducted according to the vendor’s protocol.

Capillary electrophoresis immunoblotting was performed by RayBiotech (Norcross, GA). Antibodies for TLR7 (Santa Cruz SC-57463) and calnexin (RayBiotech) were used. Each sample was loaded at 0.2 mg/mL.

### Protein markers of TLR7 activation

A list of proteins characteristically induced by TLR7 activation was obtained from Figure 9c of [13]. Quantitative MS/MS spectra were available for nine of these proteins in our previous dataset comparing four onset/AMT-hot tubers to four non-tuber controls [14]. For each protein, the difference in expression was calculated between onset/hot tubers and controls.

### Statistical analysis

Analysis was performed with JMP14. Levene’s test for equality of variance was used to compare the variance between categories. No significance in the difference of variance was detected; therefore, we assumed homogeneity of variance. The relationship of IL-23A expression to AMT uptake and seizure status was investigated using two-way ANOVA with factors specified as: AMT uptake (hot/cold) and seizure status (non-onset/onset). Since there were only two levels for each factor, a post hoc test was not needed [15]. Regression analysis of IL-23A and miR-142-3p expression was performed using least squares. The significance of change in expression for TLR7 and NF-kB target proteins was performed using one-sample *t* test of log(tuber/control) against zero (two-tailed). Results were considered significant at *P* ≤ 0.05 for all statistical tests.

## Results

**Fig. 1:**
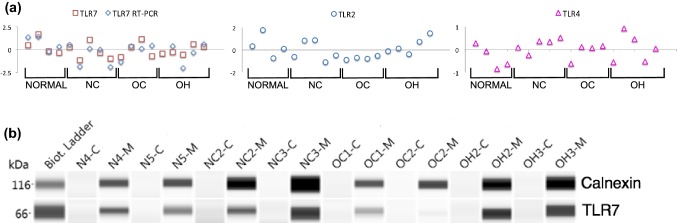
TLR7 transcript and protein are expressed in TSC cortex. **a** TLR7 transcript expression is present in all TSC specimens. Expression of TLRs 2, 4 and 7 was measured using the NanoString nCounter neuroinflammation assay and values for each specimen were normalized to the mean for the given TLR and shown in log2 scale. TLR7 was also quantified using qPCR. Expression levels were concordant between the two technologies (*P *= 0.0162, Spearman’s rho). Levels for the TLRs were not statistically significant when compared between categories. **b** Protein levels of signaling-competent TLR7 were detected in membrane fractions. Capillary electrophoresis Western analysis of membrane (M) and cytosolic (C) fractions was performed on two specimens for each tissue category. Calnexin was used as a marker specific to membrane fractions. Results are displayed as pseudo blots

Gene transcripts of TLRs 2, 4, and 7 were present in each TSC tissue specimen (Fig. 1a), but levels were not significantly associated with specimen categories: onset/AMT-hot tubers (OH); onset/AMT-cold (OC); non-onset/AMT-cold (NC); and non-tuber control (Normal/NT). Gene expression of TLR7 was analyzed using two independent technologies and results were concordant (*P *= 0.0162). TLR7 protein expression was detected using capillary electrophoresis immunoblotting of membrane and cytosolic fractions from two specimens of each category (Fig. 1b). Precursor TLR7 undergoes enzymatic proteolysis to produce a signaling-competent C-terminal fragment (~ 60 kDa) that accumulates within endosomes, and is necessary and sufficient for TLR7 receptor signaling [16–18]. We found the signaling-competent form of TLR7 in the membrane fraction of each cortex specimen, as expected for endosomal accumulation.

**Fig. 2:**
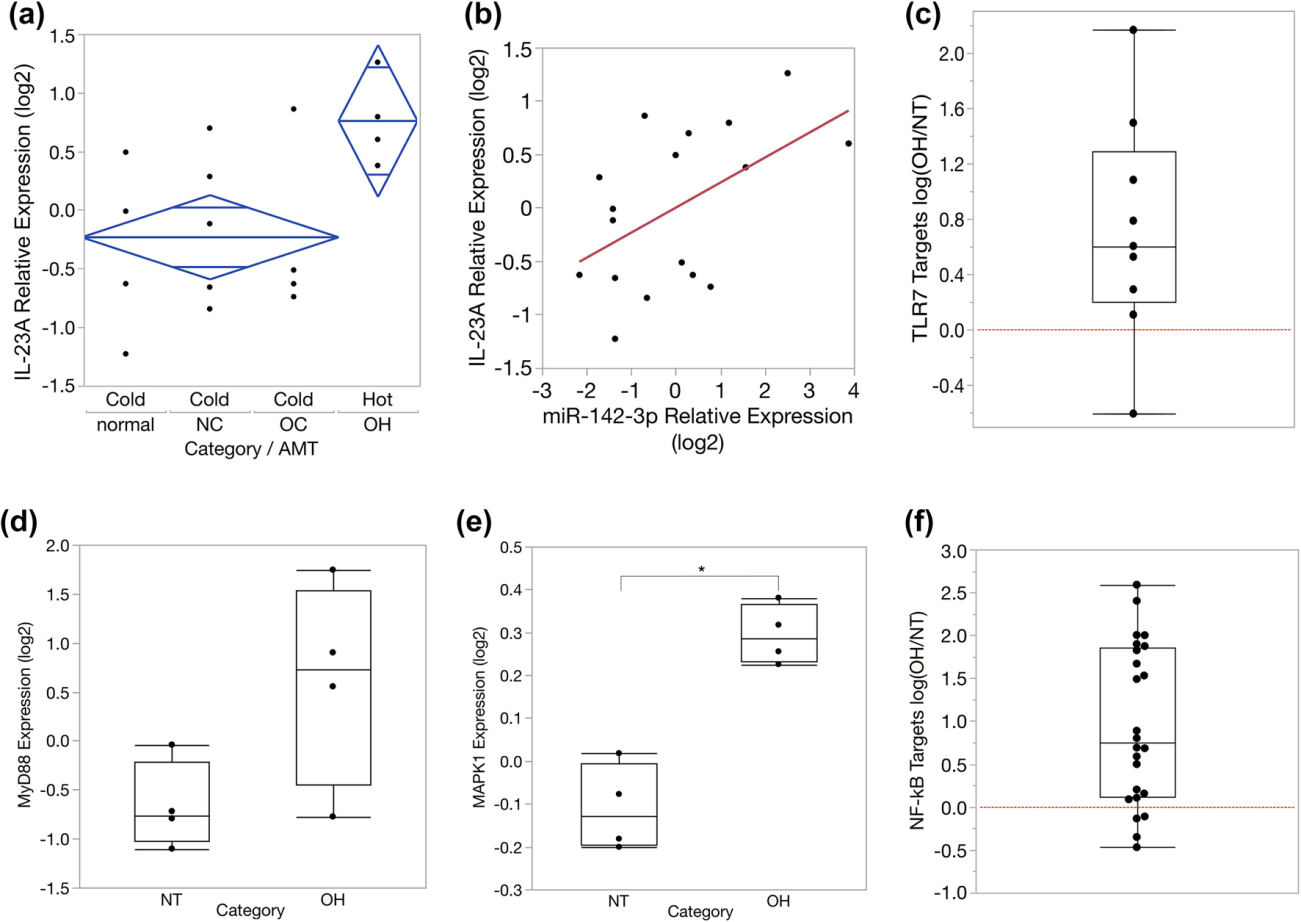
Evidence of TLR7 pathway activation in AMT-hot epileptogenic TSC tubers. **a** IL-23A expression is an established marker of TLR7 activation. Using qPCR we measured expression of IL-23A in each category. Two-way ANOVA was used to investigate the relationship of IL-23A to AMT uptake and seizure onset status. IL-23A is significantly associated with AMT uptake and increased in AMT-hot tubers (OH), *P *= 0.0387, *F* ratio = 5.206. 95% confidence intervals (diamonds) are shown for AMT hot and cold groups, with mean values and overlap marks (horizontal bars near the tips of the diamonds). Vertical separation between the overlap marks of the two diamonds indicates statistical significance. *N *= 4,5,4,4, respectively, for normal, NC, OC, and OH categories. **b** Regression analysis demonstrates that expression of miR-142-3p (a microRNA ligand and activator of TLR7) is a significant predictor of IL-23A mRNA levels in TSC tissue (*P *= 0.0318). **c** A set of proteins characteristic for TLR7 activation is significantly increased in OH tubers (*P *= 0.0283). Proteins established as a signature of TLR7 activation [13] were analyzed using our previous quantitative proteomics dataset comparing OH vs normal control (NT) [14]. MS/MS spectra were available for nine signature proteins (C1QB, TRAFD1, HSPH1, TNS3, TAPBP, PSMB9, TAP2, TRADD, TNFAIP2). The difference in expression for each protein was calculated as log(OH/NT). Positive values above the dashed line show increased expression for 8 of 9 proteins in OH specimens. **d** The median level of MyD88 protein is increased in OH tubers, albeit above statistical significance, *P *= 0.0665. **e** MAPK1 protein, downstream of TLR7, is increased in OH tubers, *P *= 0.0006. **f** A set of proteins known to be induced by NF-kB is significantly increased in OH tubers, *P *< 0.0001. This observation was originally reported in [14]. Here, we present the change in expression for each NF-kB target protein, calculated as log(OH/NT). Positive values above the dashed line indicate increased expression for 20 of 24 NF-kB target proteins in OH specimens

IL-23A expression is a marker of TLR7 activation. ANOVA analysis revealed that IL-23A is significantly associated with AMT uptake (Fig. 2a), with higher expression found in AMT-hot specimens. This observation links TLR7 activation to neuroinflammation indicated by AMT uptake. Since miR-142-3p is a known ligand and activator of TLR7 [7], we investigated if IL-23A expression in these tissues is correlated to tissue-matched miR-142-3p levels. We applied regression analysis and found that miR-142-3p is a significant predictor of IL-23A expression, and a positive correlation is evident (Fig. 2b). IL-23A is a marker of TLR7 activation, so this pairwise relationship suggests that TLR7 activation in these tissues is linked to miR-142-3p levels.

To further examine TLR7 activation in TSC tubers, we queried our previous quantitative proteomics dataset derived from a comparison of onset/AMT-hot tubers and non-tuber control tissue [14]. Here, we calculated expression changes for nine proteins that are characteristic for TLR7 activation [13]. We found that eight were increased an average of 1.6-fold in epileptogenic tubers compared to control tissue (*P *= 0.0283, Fig. 2c). All TLRs (except TLR3) induce downstream signaling through the MyD88 adapter protein and stimulate two pathways: NF-kB and mitogen-activated protein kinases (MAPK). We found that median levels of MyD88 and MAPK1 are both increased in onset/AMT-hot tubers (Fig. 2d, e), and we previously reported NF-kB activation [14]. In OH tubers, 20 of 24 proteins known to be induced by NF-kB were significantly increased in expression (*P *< 0.0001, Fig. 2f). Since TLRs 3 and 4 can cause subtle induction of IL-23A [9], we examined protein expression of two distinctive markers of TLR-3/4 activation: IFIT1 and MX1 [19]. These proteins were not significantly changed (*P *> 0.2), suggesting the observed IL-23A induction was driven by TLR7.

## Discussion

Neuroinflammation is believed to be both an outcome and a contributor to recurrent seizures, although the molecular events in this cyclical relationship are poorly understood. We previously linked increased miR-142-3p levels to inflammatory signaling in epileptogenic TSC tubers, as indicated by elevated AMT-PET uptake, yet the mechanism was unclear [6]. Another group concurrently demonstrated that miR-142-3p is a ligand and potent activator of the TLR7 receptor [7]. That finding provided an important link to other studies showing TLR7 induces IDO1 and activates the kynurenine pathway, which is responsible for AMT uptake in epileptogenic lesions. Here, we established that TLR7 is indeed expressed in TSC tubers, demonstrating the capacity to signal through this pathway. However, the extent of TLR activation cannot be reliably inferred from receptor expression levels [20, 21]. By examining characteristic downstream markers, we found evidence of TLR7 pathway activation in AMT-hot epileptogenic lesions. Importantly, IL-23A was significantly correlated to miR-142-3p levels, a known ligand and activator of TLR7.

The interaction among TLRs is complex and includes cooperative and antagonistic crosstalk. TLR7 has been shown to act synergistically with TLRs 2 and 4 [11]. Increased TLR4 expression was found in tissue of mesial temporal lobe epilepsy patients [22, 23]. Antagonistically, miR-142-3p has been shown to inhibit TLR4 activation [24], and TLR4 was reported to repress miR-142-3p expression [25]. The notably high levels of miR-142-3p in OH tubers suggest the dominant TLR pathway in this subset of lesions may be through TLR7. Further studies are needed to elucidate the relative roles of TLRs 2, 4 and 7 in the inflammatory response in epilepsy in TSC. Subsequent work may lead to opportunities for the use of neuroimaging and TLR7 inhibitors in a personalized approach to treating intractable epilepsy. Importantly, drugs are currently available to inhibit the TLR7 pathway. The antimalarial drug chloroquine and derivatives inhibit activation of endosomal TLRs and are used for treatment of lupus [26]. Chloroquine crosses the blood brain barrier (BBB), and recent work has investigated the use of chloroquine for brain malignancies [27, 28]. A number of TLR7 antagonists are in clinical trials for treatment of autoimmune and inflammatory diseases such as rheumatoid arthritis, colitis, and multiple sclerosis [29–31].
